# DNA methylation in silkworm genome may provide insights into epigenetic regulation of response to *Bombyx mori* cypovirus infection

**DOI:** 10.1038/s41598-017-16357-7

**Published:** 2017-11-22

**Authors:** Ping Wu, Wencai Jie, Qi Shang, Enoch Annan, Xiaoxu Jiang, Chenxiang Hou, Tao Chen, Xijie Guo

**Affiliations:** 1 Sericultural Research Institute, Jiangsu University of Science and Technology, Zhenjiang, Jiangsu China; 2 Beijing Genomics Institute (BGI), Shenzhen, Guangdong China

## Abstract

DNA methylation is an important epigenetic modification that regulates a wide range of biological processes including immune response. However, information on the epigenetics-mediated immune mechanisms in insects is limited. Therefore, in this study, we examined transcriptomes and DNA methylomes in the fat body and midgut tissues of silkworm, *Bombyx mori* with or without *B. mori* cytoplasmic polyhedrosis virus (BmCPV) infection. The transcriptional profile and the genomic DNA methylation patterns in the midgut and fat body were tissue-specific and dynamically altered after BmCPV challenge. KEGG pathway analysis revealed that differentially methylated genes (DMGs) could be involved in pathways of RNA transport, RNA degradation, nucleotide excision repair, DNA replication, etc. 27 genes were shown to have both differential expression and differential methylation in the midgut and fat body of infected larvae, respectively, indicating that the BmCPV infection-induced expression changes of these genes could be mediated by variations in DNA methylation. BS-PCR validated the hypomethylation of G2/M phase-specific E3 ubiquitin-protein ligase-like gene in the BmCPV infected midgut. These results demonstrated that epigenetic regulation may play roles in host-virus interaction in silkworm and would be potential value for further studies on mechanism of BmCPV epithelial-specific infection and epigenetic regulation in the silkworm.

## Introduction

Pathogens infection, silkworm strain, and climatic condition have drastic effects on the yield and quality of the silk produced. BmCPV is a major virus that specifically infects the epithelial cells of the silkworm midgut^1,2^. BmCPV is a double-stranded RNA (dsRNA) virus that belongs to the CPV subfamily within the genus Cypovirus, family Reoviridae^3^. The genome of BmCPV is composed of 10 discrete dsRNA segments among which S1-S4 and S6-S7 encode the viral structural proteins, while S5 and S8-S10 encode nonstructural proteins^4^. The infected silkworms are characterized by hypogenesis, emaciation, and sluggishness. As the infection advances, white wrinkles can be observed in the posterior part of the midgut, which is the typical symptom of CPV infection-induced disease^1^.

Studies on BmCPV-host interaction have reported as follows: (1) Gene expression profiles in the silkworm midgut changed after BmCVP infection based on its interactions with the pathogen^5–7^. For example, some genes related to immune response, such as serpin5, heat shock protein genes, amino peptidases N enzymes, were induced by BmCPV, while those genes related to metabolic pathway were down-regulated^7^. (2) Differentially expressed microRNAs are observed in BmCPV-infected silkworm midguts^8^. Cellular microRNAs and BmCPV genome encoded microRNAs could affect BmCPV replication^9,10^, suggesting that microRNAs may play important roles in BmCPV-host interaction. (3) Autophagy is only slightly induced by BmCPV infection, and could not prevent the invasion and replication of the virus^11^. (4) Establishing a BmCPV infection depends on other factors interacting with integrin beta and receptor for activated protein kinase C (RACK1) to form a receptor complex for the entry of BmCPV^12^. Thus far, the molecular mechanism underlying the midgut infection of BmCPV and the antivirus immune response to BmCPV infection is not clearly understood due to the complexity in dsRNA virus infection.

DNA methylation, one of the most important epigenetic modifications in eukaryotes, has been proven to play key roles in a broad range of biological processes including the regulation of gene expression, tumorigenesis, embryonic development, virus infection, and antiviral defenses^13–17^. Compared to vertebrates and plants, there are some unique features in the DNA methylation of insects. It is mainly mediated by DNA methyltransferases (Dnmts), which vary greatly in different insect species^18–21^. In *B. mori* only two have been reported, DNMT1 and DNMT2. BmDNMT-1 retained the function as maintenance DNMT, but its sensitivity to metal ions is different from mammalian DNMT-1^22^. In insect genomes, DNA methylation is predominant in gene bodies^23,24^ and enhances gene expression^25^. The overall level of DNA methylation in most insects is lower than 1%^24,26–28^. In insects, DNA methylation mostly regulates embryonic development, participates in genomic imprinting, regulates caste and wing differentiation, sex determination, and is involved in pesticide resistance^24,28–32^.

When the host is invaded by pathogens, gene expression is regulated via changes in DNA methylation levels or patterns, leading to the activation or suppression of relevant signaling pathways and triggering a series of immune responses against viral invasion^15^. Till date, functional analysis of DNA methylation in insect-pathogens interaction is limited. Therefore, in this study, we profiled DNA methylomes and transcriptomes in the fat body and midgut tissues of *B. mori* with or without BmCPV infection using whole-genome bisulfite sequencing (WGBS) and RNA sequencing (RNA-Seq) technologies. The correlation between gene expression and DNA methylation patterns was analyzed. This is the first report on exploring the epigenetic regulation mechanisms on the immune response of *B. mori* and our findings provide insight into the transcriptional and epigenetic mechanisms underlying the interactive responses between silkworm and pathogens.

## Results and Discussion

### BmCPV infection confirmation

BmCPV exclusively infected the epithelial cells of silkworm midguts. However, the fat body, which is the immune organ of silkworm larvae, was hardly infected by BmCPV. Infection of the midgut was confirmed by observing white wrinkles in posterior midguts and multiple polyhedrons through the microscope, while in fat bodies of infected silkworms, no polyhedron was observed. Further, quantitative real-time PCR (qRT-PCR) results revealed that the expression of BmCPV polyhedrin gene in fat bodies of infected silkworms was significantly lower than in midguts, suggesting that fat body was not infected by BmCPV (Supplementary Fig. S1).

### Transcriptional profiles of silkworm fat body and midgut are altered upon BmCPV infection

**Figure 1:**
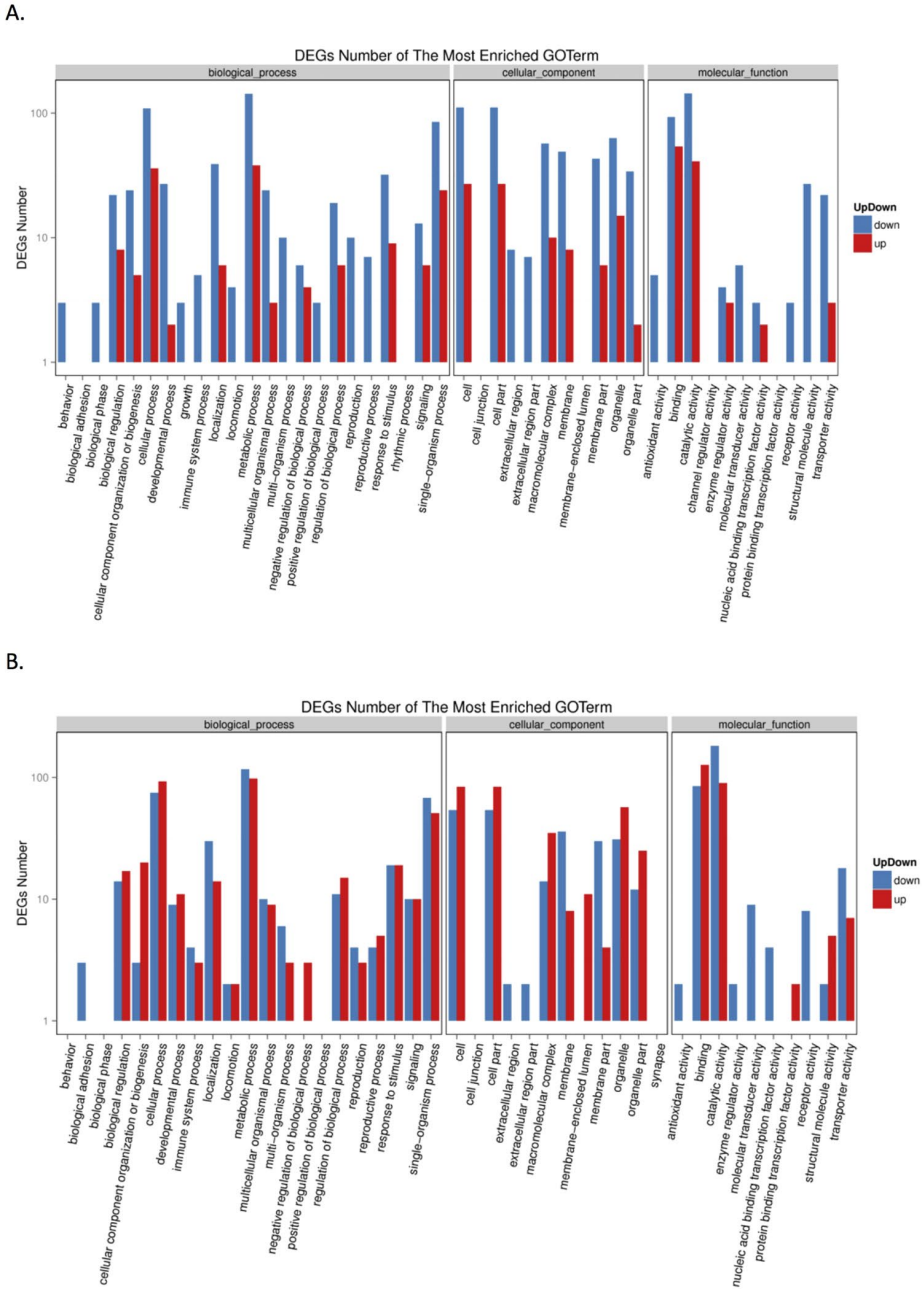
Annotation of differentially expressed genes with WEGO in fat body (**A**) and midgut (**B**). GO annotation was performed by mapping genes to GO terms in the GO database (http://www.geneontology.org). GO enrichment analysis was conducted by hypergeometric test with corrected p-value ≤ 0.05 as a threshold. Gene numbers and percentages are listed for each category.

**Figure 2:**
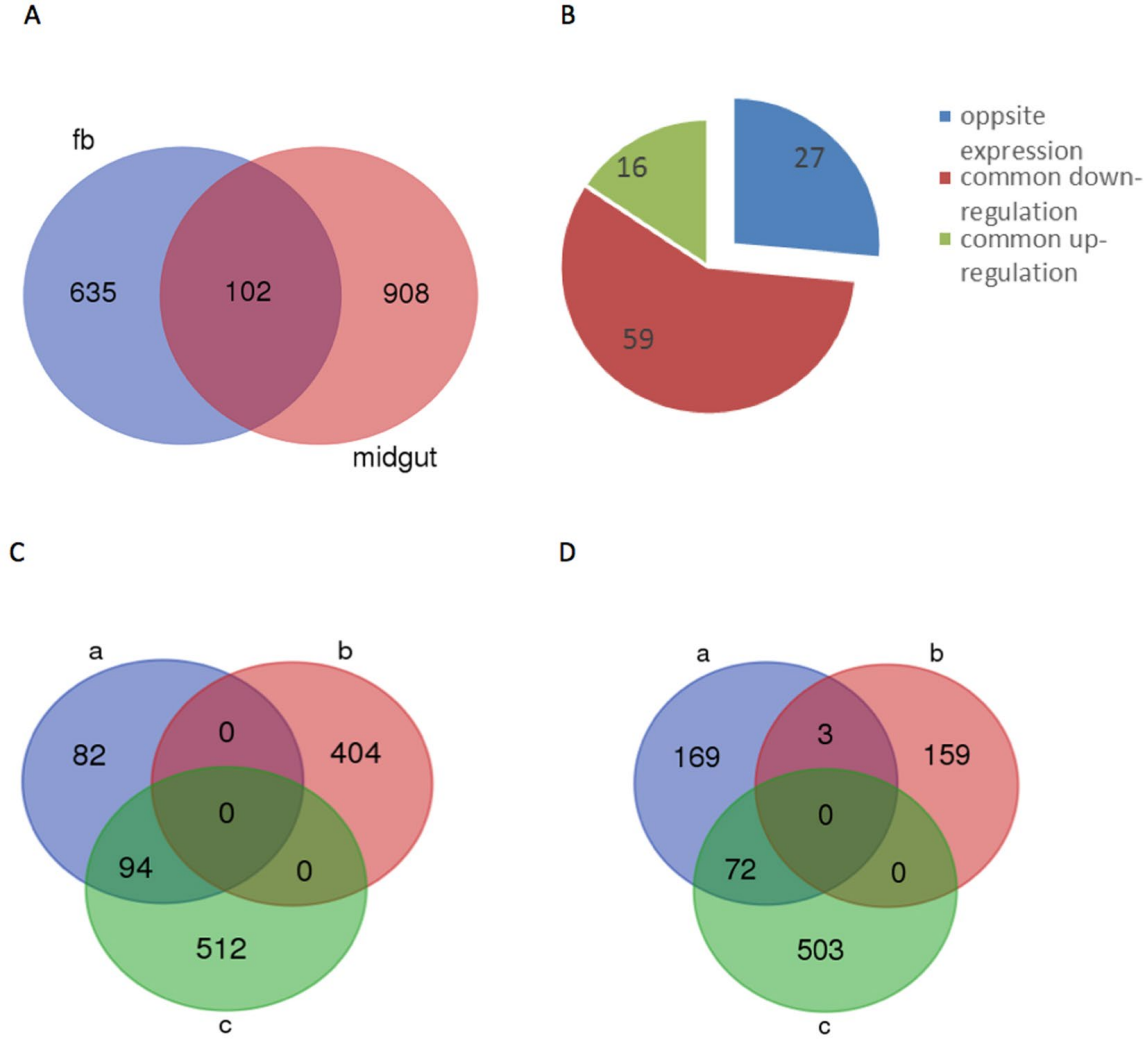
Transcriptional patterns of silkworm fat body and midgut are altered upon BmCPV infection. (**A**) Venn diagram shows 737 (left panel) and 1010 (right panel) differentially expressed genes in fat bodies and midguts of infected larvae, respectively compared to uninfected tissues. In total, 102 genes show differential expression in both fat bodies and midguts. (**B**) Among the common 102 genes, 16 are up-regulated and 59 are down-regulated. The expression pattern of 27 genes in fat bodies is opposite to that in midguts. (**C**) Venn diagram shows 94 highly or uniquely expressed genes down-regulated in midguts upon BmCPV infection. “a” represents highly or uniquely expressed genes in midguts. “b” represents up-regulated genes in infected midguts. “c” represents down-regulated genes in infected midguts. (**D**) Venn diagram shows 3 and 72 highly or uniquely expressed genes up-regulated and down-regulated in fat bodies, respectively upon BmCPV infection. “a” represents highly or uniquely expressed genes in fat bodies. “b” represents up-regulated genes in fat bodies of infected larvae. “c” represents down-regulated genes in fat bodies of infected larvae.

The mechanism of the midgut epithelia-specific infection by BmCPV is not reported yet. To explore the potential mechanism, we compared the transcriptional profiles of the midgut and fat body in control and infected silkworm larvae by RNA-Seq. More than 13,000,000 clean reads were obtained from each sample and the summary of the sequenced data is shown in Supplementary Table S1. The sequence data are deposited in the NCBI Sequence Read Archive database (http://www.ncbi.nlm.nih.gov/sra/) under the accession number PRJNA381877. Hierarchical clustering analysis suggested that midgut and fat body had distinct gene expression profiles and were clearly separated (Supplementary Fig. S2). The number of significantly differentially expressed genes in infected midgut was 1010, with 404 genes up-regulated and 606 genes down-regulated (Supplementary Table S2). In the fat body, we detected 737 differentially expressed genes, among which, the transcript level of 162 genes was increased while 575 genes was decreased in response to BmCPV infection (Supplementary Table S3). KEGG pathway analysis showed that the differentially expressed genes of both midgut and fat body tissues were involved in cellular processes, environmental information processing, genetic information processing, human diseases, metabolism and organismal systems pathways (Supplementary Fig. S3). GO analysis revealed that the down-regulated genes in the fat body were enriched in behavior, biological phase, growth, reproductive process, immune system process, receptor activity, structural molecule activity, etc. (Fig. 1A). In the midgut, we found that the genes classified to the negative regulation of biological process, protein binding transcription factor activity, and membrane-enclosed lumen were induced by BmCPV (Fig. 1B). Further, venn diagram analysis revealed 102 genes, which differentially expressed in both midguts and fat bodies of infected silkworms (Fig. 2A, Supplementary Table S4). Among them, 16 genes were up-regulated and 59 genes were down-regulated in both tissues. Notably, within the 16 up-regulated genes, 7 genes with the GO function of binding, accounted for 78% of the GO annotation, suggesting that these genes may be involved in BmCPV infection and may play similar roles or have similar effects in response to BmCPV infection in different tissues. Interestingly, we found 27 genes to have the opposite expression pattern induced by BmCPV infection (Fig. 2B). For example, the *B. mori* polyubiquitin-A-like isoform X1 gene (GeneID: 101742698) was up-regulated in fat body of infected larvae while it was significantly down-regulated in infected midgut. Transcript levels of the ribosome protein genes (GeneID: 692424, 692657, and 732854) and several immune-related genes (GeneID: 101738190, 101742015, and 101739771) was up-regulated in the infected midgut but down-regulated in fat body of the infected silkworms. We presume that the opposite expression pattern may indicate that these genes may have different roles or may be involved in different pathways response due to tissue specificity.

We compared the abundance of genes between control fat body and control midgut to determine the tissue-specific expression profile. The results revealed that about 3000 genes displayed differential expression pattern between the two tissues. We selected the genes with log2-ratio value >6.6 (the expression level of these genes in midgut was 100 times greater than in fat body) or <−6.6 (the expression level of these genes in fat body was 100 times greater than in midgut) in control fat body vs. control midgut group as highly or uniquely expressed genes. As a result, a total of 176 and 244 highly or uniquely expressed genes were identified in the midgut and fat body, respectively (Supplementary Table S5). For example, esterase-related genes (GeneID: 101738777, 101745376, 100144572, 101740845, and 692770) were highly expressed in midgut but not expressed in fat body. The genes with unique expression in the midgut or fat body may play key roles in maintaining tissue-specificity. We then explored how the expression level of these genes was changed after the BmCPV challenge. For this, venn diagrams were created with the data of differentially expressed genes and highly expressed genes. The results (Fig. 2C,D) demonstrated that in the midgut, 94 highly or uniquely expressed genes (>50%) were down-regulated after BmCPV infection (Supplementary Table S6), while in the fat body, 30% of the highly or uniquely expressed genes showed differential expression including 73 down-regulated and 3 up-regulated following BmCPV infection (Supplementary Table S6). GO analysis revealed that these down-regulated genes in the fat body were grouped into 25 categories, among which, 12 categories were unique compared to the midgut and included developmental process, enzyme regulator activity, immune system process, etc. (Supplementary Fig. S4).

Changes in transcript levels of host genes appear to be a common consequence of a viral infection^7^. Comparing the transcriptome of midgut with that of fat body, we can get several features: (1) Although BmCPV hardly infects the fat body, more than 700 genes were observed to have differential expression and 80% accounted for down-regulation (Supplementary Table S3). The proportion of down-regulated genes was higher than that of midgut (60%). (2) Among 102 genes, which differentially expressed in both midguts and fat bodies of infected silkworms, there were 27 genes to have the opposite expression pattern in different tissue induced by BmCPV infection. (3) Most of those DEGs, (>90%), which are highly or uniquely expressed in midgut or fat body were all down-regulated after BmCPV infection and the functions of these DEGs are diverse. These features suggest that the midgut and fat body have distinct expression profiles. The molecular mechanisms against BmCPV may be different between the two tissues. Large quantity of down-regulated genes were detected during the late stage of BmCPV infection (96 h), suggesting that the expression patterns of genes related to BmCPV infection were reorganized for anti-viral responses to diminish the negative effects of viral invasion or to facilitate viral proliferation and avoid host defense triggered by virus.

We could not however, clearly demonstrate the molecular mechanism for epithelia-specific infection by BmCPV. Supplementary Table S4–S6 provide a lists of candidate genes for future functional characterization. We will particularly focus on the functions of genes involved in membrane binding, trans-membrane transduction, receptor activity, and immune response by using transgenesis and RNAi technologies.

### Identifying changes in DNA methylation patterns in the host cell genome after infection with BmCPV

**Figure 3:**
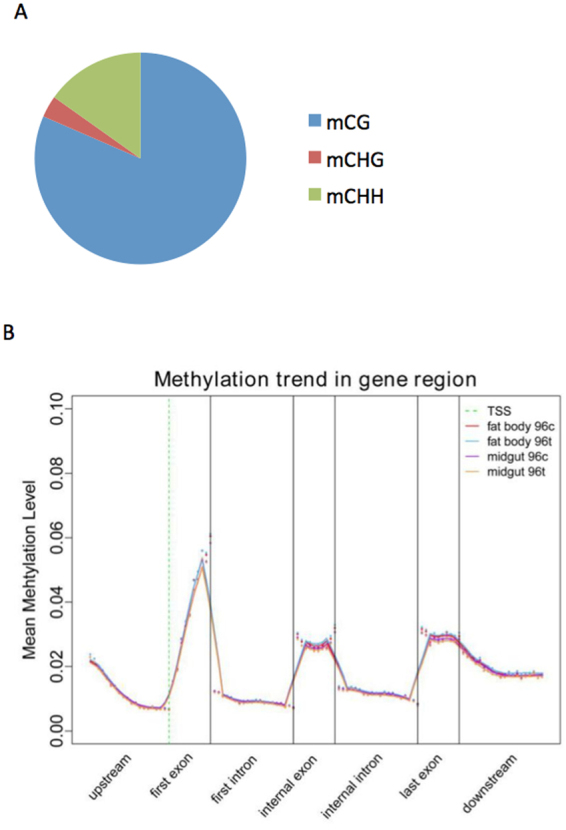
Average DNA methylation level and the distribution in the whole genome in four samples. (**A**) The average proportion of different types of methylated cytosines in the four samples. (**B**) Distribution of DNA methylation levels of genes in four samples. The gene structure is defined by seven different features, denoted by the x-axis. The length of each feature was normalized and divided into equal numbers of bins. Each dot denotes the mean methylation level per bin and the respective lines denote the 5-bin moving average. Each feature was analyzed separately for the numbers listed in the table below the figure. The green vertical line indicates the mean location of the transcription start sites.

Alternations in global DNA methylation patterns due to pathogen invasion have been widely reported in mammals and plants^14,33–35^. To evaluate epigenetic changes in insect cells caused by BmCPV infection, methylation of genomic DNA was determined in four samples, namely fat body 96t, midgut 96t (BmCPV infected), fat body 96c, and midgut 96c (BmCPV uninfected) using bisulfite sequencing. We observed that about 0.6% of genomic cytosines are methylcytosines, and that 81% of these were CG dinucleotides (Fig. 3A). Xiang *et al*. reported that non-CG mCs are either nonexistent or very rare in the silkworm^25^. Here, we found about 19% mCs at CHG and CHH sites (H = A, C or T), further study need to be carried out for verifying the true or false positive. The average methylation level at CG locations in the whole genome was about 1%, which is significantly higher than 0.67% in the silk glands of silkworm^25^. In order to understand the relationship between DNA methylation profiles and gene expression, DNA methylation profiles were divided into seven distinct genomic functional regions to study the changes in methylation levels (Fig. 3B). In agreement with previous studies on other insects^23–25,27^, we found that in silkworm DNA methylation is mostly targeted to gene bodies. DNA methylation levels peaked in the first exon and sharply decreased in intron regions, with 3′ downstream region showing more methylation than the 5′ upstream region. This result is similar to the pattern reported in other insects^25,28,33^. Fractional methylation levels in gene regions exhibited a different pattern between infected and non-infected samples. Genomic DNA methylation levels of each of the four samples were distinct as shown in Supplementary Fig. S5, suggesting that DNA methylation is tissue-specific and dynamically responds to stimuli.

### Differential DNA methylation genes (DMGs) in response to BmCPV infection

**Figure 4:**
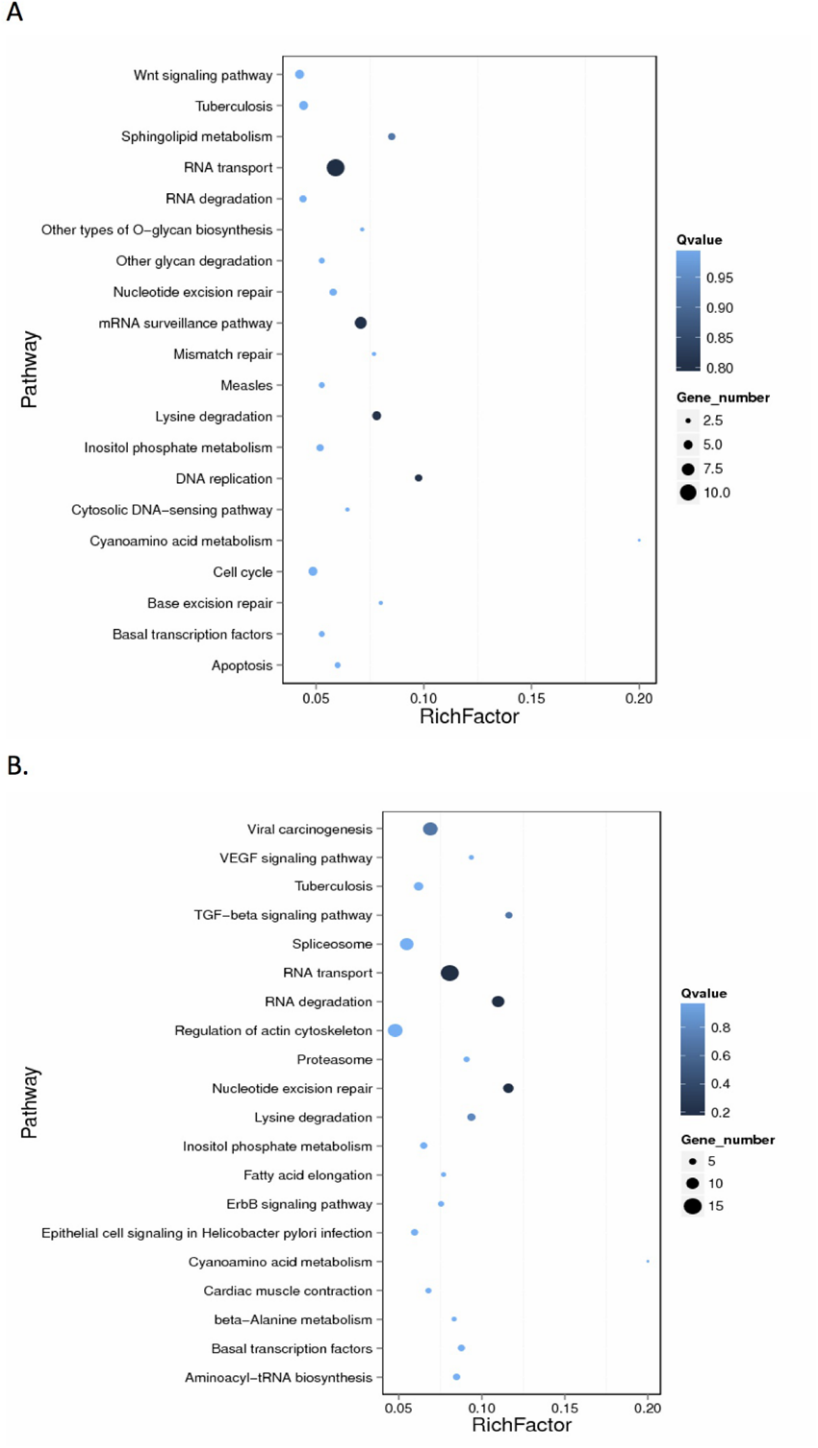
KEGG pathway enrichment of differentially methylated genes following BmCPV infection. (**A**) Differentially methylated genes in infected midguts. (**B**) Differentially methylated genes in fat bodies of infected larvae. RichFactor is the ratio of the number of differentially expressed genes in this pathway term to the number of all genes in this pathway term. Greater RichFator means greater intensiveness. Qvalue is corrected p-value ranging from 0–1, and less Qvalue means greater intensiveness. Only the top 20 of the enriched pathway terms are displayed here.

DNA methylation is reported to be involved in antiviral responses^15,17^. To explore the potential function of DNA methylation in insect immune response, we compared the DMR of the whole genome between BmCPV infected midgut or fat body with their corresponding uninfected control tissues. The results revealed that 394 DMRs were present in the midgut 96t vs. midgut 96c group with 271 located in the gene region. Among these, 119 were hypermethylated and 151 genes were hypomethylated (Supplementary Table S7). In the fat body, 591 DMRs were observed and 410 were associated with genes, among which 223 genes were hypermethylated and 187 were hypomethylated (Supplementary Table S8). KEGG pathway analysis of DMGs revealed that DMGs were involved in pathways of RNA transport, RNA degradation, nucleotide excision repair, DNA replication, etc. (Fig. 4), suggesting that variation in DNA methylation could regulate the expression of genes involving in multiple important pathways.

### Effects of DMR on gene expression level in silkworm after infection with BmCPV

To determine whether the changes in CG methylation observed in silkworm following BmCPV infection altered gene expression, we synthetically analyzed the data of DEGs and DMRs. As a result, we found 27 DMGs showing differential mRNA levels in the fat body of the infected silkworm (Table 1) and midgut, respectively (Table 2), indicating that these genes related to BmCPV infection may change their expression level via variation in DNA methylation. Analysis of the distribution of DNA methylation on these genes revealed that CG methylation in all 27 genes in the midgut occurred in the gene body, predominantly in the introns of genes (Supplementary Fig. S6). However, in the fat body, DNA methylation was observed in the 5′ or 3′ UTR regions of 6 genes. Both in midgut and fat body, there were parts of genes, whose intron-extron boundaries were also DNA methylated, suggesting that in *B. mori* DNA methylation is likely associated with alternative splicing^36,37^.

**Table 1:** Gene lists of both DMGs and DEGs in fat bodies with BmCPV infection.

Gene ID	log2Ratio of DEGs (fat_body_96t/fat_body_96c)	PPEE	log2Ratio of DMGs (fat_body_96t/fat_body_96c)	Annotation
101738991	1.4241	9.36E-04	−1.0340	heterogeneous nuclear ribonucleoprotein 87F-like [Bombyx mori]
732899	1.3718	1.34E-11	1.0000	nascent polypeptide associated complex protein alpha subunit [Bombyx mori]
101746243	1.3248	7.69E-03	1.2630	protein KRI1 homolog [Bombyx mori]
101742308	1.2914	7.67E-04	1.0510	isoleucine–tRNA ligase, cytoplasmic-like [Bombyx mori]
101746886	1.1813	4.92E-04	1.1600	set1/Ash2 histone methyltransferase complex subunit ASH2-like [Bombyx mori]
692990	1.1672	4.94E-02	1.1220	DnaJ (Hsp40) homolog 2 [Bombyx mori]
101739290	1.1455	3.88E-03	−1.0000	inhibitor of Bruton tyrosine kinase-like [Bombyx mori]
101744580	1.0709	5.84E-04	1.0000	hypothetical protein KGM_18067 [Danaus plexippus]
692956	1.0531	2.27E-06	−1.3790	nucleoplasmin isoform 2 [Bombyx mori]
101736883	1.0098	3.21E-07	−1.0540	protein DEK-like isoform X1 [Bombyx mori]
101738436	1.0063	8.12E-07	−1.6370	splicing factor 3B subunit 1-like [Bombyx mori]
101741560	−1.0214	9.57E-06	−1.6440	uncharacterized protein LOC101741560 [Bombyx mori]
101737841	−1.0766	6.68E-04	1.3540	acetyl-CoA hydrolase-like [Bombyx mori]
693058	−1.0958	1.20E-13	1.4590	ribosomal protein S16 [Bombyx mori]
101745599	−1.1041	4.56E-03	−1.2220	enoyl-CoA hydratase precursor 1 [Bombyx mori]
733116	−1.1306	0.00	−20.0000	eukaryotic translation initiation factor 3 subunit F [Bombyx mori]
101743877	−1.1365	0.00	1.1520	hypothetical protein KGM_08776 [Danaus plexippus]
101746182	−1.2844	2.22E-16	1.4950	uncharacterized protein LOC101746182 [Bombyx mori]
101738186	−1.4037	1.48E-05	1.2220	aconitate hydratase, mitochondrial-like [Bombyx mori]
732876	−1.4106	0.00	−1.0930	proteasome 25 kDa subunit [Bombyx mori]
100134921	−1.4995	2.73E-02	1.2060	ubiquinol-cytochrome c reductase [Bombyx mori]
101738009	−1.6180	1.41E-05	1.1150	ATP synthase lipid-binding protein, mitochondrial;
101738244	−1.6892	2.21E-02	−1.0000	putative Serine/threonine-protein kinase WNK3 [Danaus plexippus]
733056	−1.7736	4.38E-07	−1.0000	putative alcohol dehydrogenase [Bombyx mori]
100862843	−1.7892	0.00	1.9180	UDP-glycosyltransferase, UGT41 A1recursor [Bombyx mori]
101744219	−1.9049	0.00	1.0700	glycosyltransferase-like protein LARGE1-like [Bombyx mori]
101735659	−1.9256	0.00	2.3220	glycine-rich cell wall structural protein 1.0-like [Bombyx mori]

**Table 2:** Gene lists of both DMGs and DEGs in midguts with BmCPV infection.

Gene ID	log2Ratio of DEGs(midgut_96t/midgut_96c)	PPEE	log2Ratio of DMGs (midgut_96t/midgut_96c)	Annotation
101735936	1.6258	5.30E-08	−1.2730	uncharacterized protein LOC101735936 [Bombyx mori]
101735989	1.8947	1.26E-13	−1.0750	RNA-binding protein 28-like [Bombyx mori]
101736907	1.7464	3.63E-03	−1.0000	uncharacterized protein LOC101742081 [Bombyx mori]
101737128	3.0148	0.00	1.0440	nucleolar protein 56-like [Bombyx mori]
101737417	1.9544	2.45E-03	1.7000	lisH domain-containing protein C1711.05-like [Bombyx mori]
101739208	2.1410	9.18E-06	−1.0700	G2/M phase-specific E3 ubiquitin-protein ligase-like [Bombyx mori]
101739510	−1.5049	0.00	−1.3920	testin-like [Bombyx mori]
101740026	1.9136	2.87E-04	−1.5360	nucleoporin NUP188 homolog [Bombyx mori]
101740154	1.8013	1.88E-04	−1.2220	telomere-associated protein RIF1-like [Bombyx mori]
101740265	1.2050	2.21E-02	−1.2020	structural maintenance of chromosomes protein 1A-like isoform X1 [Bombyx mori]
101740584	1.5595	3.45E-11	1.1070	cleavage and polyadenylation specificity factor subunit CG7185-like isoform X1 [Bombyx mori]
101741392	2.2260	2.41E-02	1.3920	glucosylceramidase-like [Bombyx mori]
101741887	2.2111	1.63E-03	1.3440	exonuclease 1-like [Bombyx mori]
101742216	−1.1865	3.22E-05	−1.0420	phosphatidylinositol 4-phosphate 3-kinase C2 domain-containing subunit alpha-like [Bombyx mori]
101742628	1.3424	0.00	−1.0000	splicing factor 3B subunit 2-like [Bombyx mori]
101742901	1.0502	9.80E-07	−1.4150	importin subunit beta-1-like [Bombyx mori]
101744992	−1.1445	1.98E-02	1.9330	apoptosis-stimulating of p53 protein 2-like [Bombyx mori]
101745449	1.3282	0.00	4.3920	protein lingerer-like [Bombyx mori]
101746062	−1.5040	0.00	−1.2900	nucleobindin-2-like isoform X1 [Bombyx mori]
101746099	1.2062	0.00	−1.3220	zinc finger RNA-binding protein-like isoform X1 [Bombyx mori]
101746131	−1.7917	3.33E-16	−1.1380	multiple coagulation factor deficiency protein 2 homolog isoform X2 [Bombyx mori]
692502	1.2292	0.00	1.4150	casein kinase 2 alpha subunit [Bombyx mori]
692744	1.2030	7.67E-04	−1.0000	50 kDa lectin [Bombyx mori]
692884	−1.0291	3.12E-02	−1.2020	2-hydroxyphytanoyl-CoA lyase [Bombyx mori]
692945	−2.5874	3.44E-11	−1.2090	kazal-type proteinase inhibitor precursor [Bombyx mori]
692978	3.3555	1.33E-05	4.8070	thymidylate synthase isoform 2 [Bombyx mori]
733031	−1.0130	4.80E-05	1.1070	vacuolar ATP synthase subunit H [Bombyx mori]

**Figure 5:**
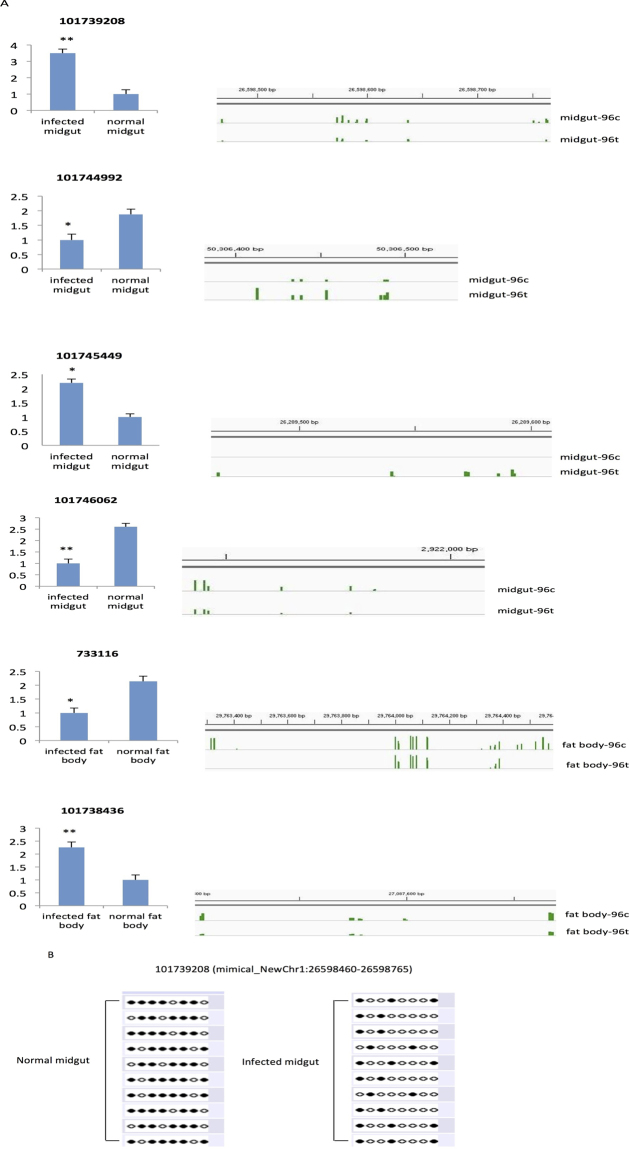
Both differentially expressed and differentially methylated genes in response to BmCPV infection. (**A**) Six genes were identified to be differentially expressed in midguts or fat bodies upon BmCPV infection by qRT-PCR on left side. RNA was extracted from midguts and fat bodies with or without BmCPV infection. Data represent the relative transcript levels of genes in the infected midguts or fat bodies compared to the control tissues from three independent samples. The error bars indicate standard deviations (**p < 0.01, *p < 0.05). The visualized image of differential methylation genes (right side) was generated by Integrative Genomics Viewer (IGV). The identification of methylated regions was performed by CpG_MPs (v1.1.0) with the required sequencing depth great than 10 and average C methylation ratio great than 0.55, and then converted to wig format by perl script for visualization on IGV. The location of green bars in the images indicates the methylated sites and the height of green bars represents the methylation levels. (**B**) Bisulfite sequencing validation of differentially methylated 101739208 in normal verse infected midgut. Targeted bisulfite sequencing validation of a region overlapping the DMR is indicated by the rectangle over the genome browser tracks. Dark circles indicate methylated and open circles mean unmethylated cytosines. Each row consists of a single sequenced clone.

CG methylation in gene body is reported to have positive correlation with gene expression levels^25,38^. In this study, we found that the effects of DNA methylation in gene body could have a negative or positive effect on gene expression following BmCPV infection. Six genes were selected for mRNA level quantification by using qRT-PCR. The results obtained were consistent with RNA-Seq analysis (Fig. 5A). Moreover, the visual images for DNA methylation level and location in the genome of these six genes were also made by IGV (Version 2.3.83) (Fig. 5A).

In this study, we found that 101739208, a homolog of G2/M phase-specific E3 ubiquitin-protein ligase-like (G2E3), was up-regulated and hypomethylated in the midgut after BmCPV infection (Fig. 5B). G2E3 is an ubiquitin ligase (E3) that is essential for early embryonic development to prevent apoptotic death. It shuttles between the cytoplasm and nucleus, concentrating in the nucleoli and relocalizing to the nucleoplasm in response to DNA damage^39,40^. Apoptosis is an effective strategy used by insects to defend themselves against pathogen invasion. Loss of G2E3 triggered apoptosis and decreased proliferation of cancer cells^41^. On the other hand, up-regulation of G2E3 suppressed apoptosis progress and helped effective infection of BmCPV in the midgut. It is suggested that in silkworm, pathogens may successfully establish infection by manipulating the expression of host genes related to the apoptosis pathway via DNA methylation modification.

Moreover, the mRNA levels (p < 0.0001) of SF3B1 (GeneID: 101738436) and SF3B2 (GeneID: 101742628) were increased significantly in BmCPV infected midgut and fat body as detected in this study. In addition, hypomethylation was observed in the introns of SF3B1 and SF3B2 genes. The SF3B1 gene encodes subunit 1 of the splicing factor 3b, which is important for anchoring the spliceosome to precursor mRNA. Numerous studies have indicated that aberrant splicing patterns or mutations in spliceosome components, including SF3B1, SF3B3, and SF3B4, are associated with cancer and tumorigenesis and can act as potential anticancer agents^42–44^. Depletion of endogenous SF3B1 abrogated the apoptotic effects^42^. We presume that hypomethylation of SF3B1 and SF3B2 may increase the transcript levels of SF3B1 and SF3B2, which may play important roles in changing the splicing pattern, and attenuating the infectivity by apoptotic cells.

Hypermethylation and down-regulation of apoptosis stimulating protein, p53-2 (ASPP2, GeneID: 101744992), were also observed in BmCPV infected midgut. P53 is a central apoptotic regulator^45^, and ASPP2 is a damage-inducible p53-binding protein that enhances apoptosis through p53-dependent and p53-independent pathways^46^. Attenuation of ASPP2 is caused by hypermethylation of the promoter and 5′ UTR regions in native leukemia blasts^47^. The hypermethylation and down-regulation of ASPP2 in BmCPV infected midgut suggests that BmCPV infection may lead to hypermethylation of ASPP2, which in turn may suppress the expression of ASPP2 and facilitate the proliferation of infected cells.

Significant up-regulation of lingerer gene (Lig, GeneID: 101745449) in infected midgut were also detected (P = 0) in this study. Lig, an UBA domain-containing protein, interacts with the RNA-binding proteins FMR1, rasputin and caprin to restrict tissue growth in *Drosophila melanogaster* ^48^. Moreover, Lig functions as a critical growth suppressor to control organ size via bantam (ban) microRNA and Hippo signaling in *D. melanogaster* ^49^. Loss of Lig increased organ size and upregulated ban and the expression of Hippo pathway target genes, while overexpression of Lig diminished ban expression and reduced organ size reduction. Previously, we identified the down-regulation of bmo-bantam in BmCPV infected midgut^8^. Here, the significant up-regulation of Lig gene in infected midgut suggests that suppression of bmo-bantam may retard the growth of infected silkworm by increasing the expression of Lig gene. Notably, we observed hypermethylation of Lig gene in BmCPV infected midgut, indicating that the two epigenetic models, microRNA as well as DNA methylation may co-regulate the expression of Lig gene to regulate maldevelopment and undergrowth of BmCPV infected larvae.

Obvious down-regulation (P = 0) and significant hypomethylation of the subunit F of eukaryotic translation initiation factor 3 (eIF3f, GeneID: 733116) was observed in the fat bodies of infected silkworm larvae. eIF3f plays important roles in cell growth in human, yeast and Arabidopsis^50^. In addition, eIF3F can stabilize p53 and interact with CLU protein, a secretory heterodimeric glycoprotein thus leading to cancer cell proliferation in humans functioning as a CLU inhibitor^51^. The overexpression of eIF3f retarded cancer cell growth and induced apoptosis^51^. Accordingly, the obvious down-regulation (p = 0) and significant hypomethylation of eIF3f in fat bodies of infected silkworm larvae indicates that eIF3f may act as a potential gene that responds to BmCPV infection.

Nucleobindin 2 (GeneID: 101746062) was down-regulated and hypomethylated in BmCPV infected midguts. Nucleobindin 2 has been demonstrated to play critical roles in tumorigenesis and tumor development in breast cancer^52^. It can enhance cell migration and invasion in breast cancer, prostate cancer, and colon cancer cells^53^. Reduction in nucleobindin-2 expression inhibited EGF-stimulated MAPK kinase/Erk phosphorylation, cell proliferation^54^ and abrogate insulin-stimulated GLUT4 translocation^55^. In this study, the down-regulation and hypomethylation of nucleobindin-2 gene in BmCPV infected midgut suggests that the host may decrease expression level of some genes responsive to BmCPV infection by changing their DNA methylation level to resist the virus infection. Interestingly, another gene, Kazal-type proteinase inhibitor precursor (GeneID: 692945), which plays important roles in physiological processes such as development and immune response^56,57^ showed the same expression pattern with 101746062, with down-regulated mRNA level and hypomethylation in infected midguts.

In total, 176 and 244 genes were highly or uniquely expressed in the midgut and fat body, respectively. Protein sequences of genes targeted by high levels of methylation are conserved relative to genes lacking methylation. Genes that are methylated in all four invertebrate taxa (honeybee, silkworm, sea squirt, and sea anemone) are enriched for housekeeping functions related to transcription and translation^58^. The highly or uniquely expressed genes in midgut and fat body detected in this study may be related to tissue-specificity and play distinct roles in tissue function. We analyzed the methylation status of these genes and the results revealed that except four genes (GeneID: 101739540, 101743290, 101737724, and 692564) in fat body, others were all unmethylated or hypomethylated, indicating that DNA methylation is preferentially targeted towards genes with ubiquitous expression, whereas the loss of DNA methylation occurred in genes with tissue-specific functions.

In summary, this is the first report on characterizing genome-wide gene expression and DNA methylation patterns associated with BmCPV infection in a model insect, *B. mori*. Both the genome-wide transcription profile and DNA methylation patterns of silkworm display tissue-specificity, which is altered upon BmCPV challenge. The rate of genomic CG methylation in the midgut and fat body is much higher than in the silk gland. CG methylation is predominantly in gene bodies, and could either positively or negatively regulate gene expression. Both gene expression level and DNA methylation level can be changed upon BmCPV infection. The mechanism of alteration of gene expression by BmCPV invasion is complicated and not clear until now. It may be regulated probably by non-coding RNA, histone modification and so on. Here, we found 27 DEGs were also DMGs, suggesting that expression level of these genes may be changed via variation in DNA methylation. It also indicated that DNA methylation may be play roles in host-pathogen interaction in insects. Further experiments need to be carried out for demonstrating how the DNA methylation would mediate the expression of important genes involved in response to BmCPV infection. On the other hand, we also found most part of DMRs do not correspond to any DEGs, which may imply that DNA methylation could also be an independent molecular pathway in response to viral infection in *B. mori*.

Our study is a valuable resource to explore the underlying mechanism of tissue-specific infection of BmCPV, and adds epigenetic modification as a new dimension to host-pathogen interaction in insects. Further experiments need to be carried out for demonstrating how the DNA methylation would mediate the expression of important genes involving in BmCPV infection.

## Materials and Methods

### Silkworm strain and virus inoculation

Domesticated silkworm strain 4008, European monovoltin and susceptible to BmCPV, was used in this study. They were reared at 25 °C, 80 ± 5% relative humidity under a photoperiod of 12 h of light and 12 h of dark up to fourth molting. Each larva was inoculated about 1 × 10^6^ BmCPV polyhedrons by oral infection. The detailed process about BmCPV inoculation was described in our previous study^8^.

### Confirmation of virus infection

Virus infection was confirmed in the following manners: (1) inoculated silkworm larvae displaying the typical symptom of white wrinkles on the posterior midgut, (2) observation of polyhedra under a microscope, and (3) presence of the BmCPV polyhedrons gene by qRT-PCR.

### Sample preparation for RNA-Seq and WGBS

The midgut and fat body of both BmCPV-infected and control larvae (uninfected) were collected at 96 h post-inoculation by dissecting the larvae on ice. Isolated tissues were then quickly rinsed in 0.8% diethylpyrocarbonate (DEPC)-treated physiologicsaline solution and frozen in liquid nitrogen and powdered. Then, the powdered samples in both infected and control groups were individually pooled into three groups for biological replication.

Half of the sample from each group was used to extract the total RNA by using the Trizol reagent (Invitrogen, USA) and for RNA-Seq assay. DNA was isolated from the other half by using DNeasy Blood and Tissue Kit (Qiagen, Germen) and for WGBS assay.

### Transcriptome analysis

RNA-Seq experiment was performed by Beijing Genomics Institute (Shenzhen, China). The process is described briefly as follows: firstly, the mRNA was enriched by using the oligo (dT) magnetic beads and fragmented at an elevated temperature. The double strand cDNA was synthesized and purified for each targeted fragments (200–500 bp). Then, the fragments were ligated to sequencing adaptors and enriched by PCR amplification. When the necessary quality control steps were passed, the library products were sequenced via Illumina Hiseq. 4000 platform. Raw reads were filtered into clean reads after removing adaptor sequences, reads consisting more than 10% unknown nucleotides, and low quality reads (more than 20% base Q ≤ 20). Clean reads were mapped to silkworm mRNA (*B. mori* assembly ASM15162v1) using Bowtie2 (version 2.2.5)^59^. Differentially expressed genes were identified by EBseq (version 1.4.0)^60^ with the screened criteria as fold change ≥ 2 and PPEE ≤ 0.05. Gene ontology (GO) annotation was performed by mapping genes to GO terms (http://www.geneontology.org), and GO enrichment analysis of differentially expressed genes (DEGs) was conducted by hypergeometric test based on ‘GO::TermFinder’ (http://www.yeastgenome.org/help/analyze/go-term-finder). The calculated p-value was processed by using Bonferroni Correction and the corrected p-value ≤ 0.05 was used as threshold. KEGG pathway annotation and enrichment analysis were performed similar to GO analysis.

### Whole genome bisulfite sequencing

For whole-genome bisulfite sequencing (WGBS), the DNA was fragmented by sonication using a Bioruptor (Diagenode, Belgium) to a mean size of approximately 250 bp, followed by adding dA to 3′ end by bluntend cloning and ligating methylated adaptors. Ligated DNA was bisulfite converted using the EZ DNA Methylation-Gold kit (ZYMO, USA). Different lengths of DNA fragments were excised from a 2% agarose gel and purified and amplified by PCR. Finally, sequencing was performed using the Illumina Hiseq. 4000 platform.

After filtering, the clean data was mapped to the silkworm genome (*B. mori* assembly ASM15162v1) by using the whole genome bisulfite sequencing mapping program (BSMAP, v2.74)^61^. Then, the mapping rate and bisulfite conversion rate was calculated for each sample.

The methylation level was determined by dividing the number of reads covering each mC by the total number of reads covering that cytosine, which was also equal to the mC/C ratio at each reference cytosine^62^.

### Identification of differentially methylated regions (DMRs)

Putative DMRs were identified by comparing methylomes from the infected and control tissue using windows that contained at least 5 CpG sites based on the following standards: (1) the whole length of DMRs is ≥ 200 bp; (2) the average methylation rate is >0.2; (3) putative DMRs has 6 × sequencing coverage; and (4) 2-fold change in methylation level and Fisher test p value ≤ 0.05. In addition, two nearby DMRs were considered as one continuous DMR if the genomic region from the start of an upstream DMR to the end of a downstream DMR also had 2-fold methylation level with a p value <= 0.05. The final dataset of DMRs was made with those that were independent from each other. GO and KEGG pathway analysis of genes containing DMRs were performed as described in the “*Transcriptome analysis*” section.

Methylated regions were identified by CpG_MPs (v1.1.0)^63^ with the required sequencing depth great than 10 and average C methylation ratio greater than 0.55, and then converted to wig format by using perl script for visualization on IGV (Integrative Genomics Viewer)^64^.

### qRT-PCR Analysis

The total RNA for RNA-Seq analysis was also used for qRT-PCR. qRT-PCR was performed according to the manufacturer’s instructions of the SYBR Premix Ex TaqTM Kit (TaKaRa, China) and run on ABI 7300 machine (Applied Biosystems, USA) with thermal cycling parameters at 95 °C for 30 s followed by 40 cycles of 95 °C for 5 s, 60 °C for 31 s. Following amplification, melting curves were constructed. Three independent experiments with three technical replicates were performed. Data were analyzed and normalized to control gene transcript levels. A relative quantitative method (2^−ΔΔCt^) and the student’s t test were used to evaluate relative expression differences. All of the primers were listed in Supplementary Table S9.

### Bisulfite-PCR validation of DMRs

Genomic DNA from infected and control tissues was prepared for bisulfite conversion as described above. Bisulfite converted DNA was amplified by PCR with Premix Ex Taq™ Hot Start Version (TaKaRa, Japan) according to the manufacturer’s instructions. Nested primers for BS-PCR were designed using the online MethPrimer program (S9 Table). PCR products were purified and cloned into the pMD19-T vector (TaKaRa, Japan). Ten different clones were then sent to Sangon Biotech Co., Ltd. (Shanghai, China) for sequencing. DNA methylation of individual CpG sites was analyzed using Quma software (http://quma.cdb.riken.jp/).

## Electronic supplementary material

Supplementary figures

Supplementary talbes
