# Supplementary material for: DNA methylation in silkworm genome may provide insights into epigenetic regulation of response to Bombyx mori cypovirus infection
Source: Sci Rep. 2017 Nov 22;7:16013. doi: 10.1038/s41598-017-16357-7 (PMC5700172; doi:10.1038/s41598-017-16357-7)
Supplement: Supplementary file 1 — Supplementary figures [file 41598_2017_16357_MOESM1_ESM.docx]

**DNA methylation in silkworm genome may** **provide insights into epigenetic regulation of response to *Bombyx mori* cypovirus infection**

Ping Wu^1*^, Wencai Jie^2*^, Qi Sang^1^, ENOCH ANNAN^1^, Xiaoxu Jiang^1^, Chenxiang Hou^1^, Tao Chen^1^, Xijie Guo^1🖂^

**Fig. S1 Validation of non-infection of fat bodies with BmCPV by qRT-PCR.** RNA was extracted from midguts and fat bodies with BmCPV infection. The data represent the relative transcript level of BmCPV polyhedrin gene in midgut or fat body in infected larvae for three independent samples carried out in triplicate. The error bars indicate standard deviations.


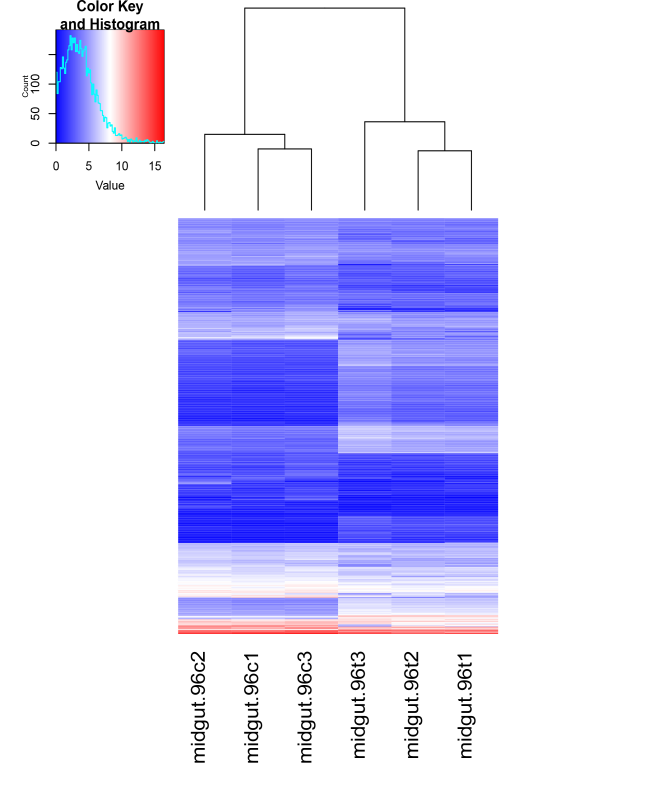

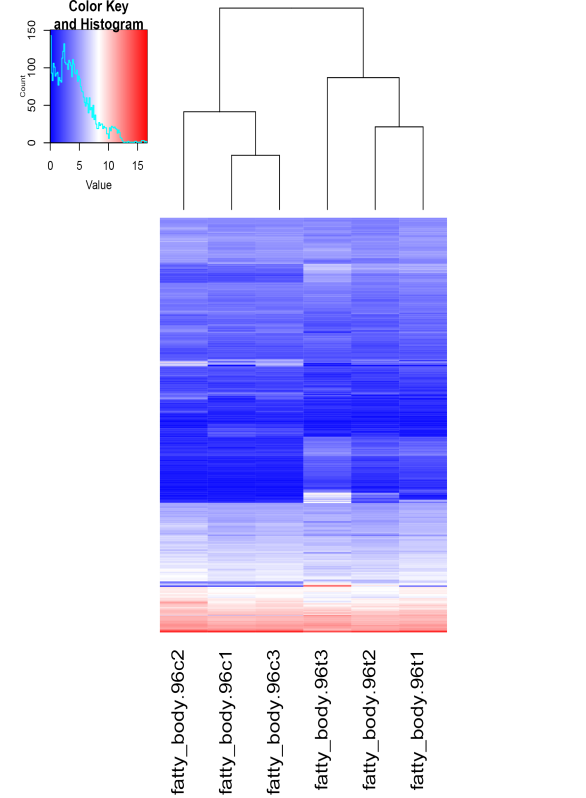


**Fig. S2 Hierarchical clustering analysis of differential expressed genes following with BmCPV.** Heatmap displays the expression changes of differentially expressed genes in fat bodies and midguts samples. Hierarchical clustering analysis identified 4 main clusters: midgut 96c, midgut 96t, and fat body 96c and fat body 96t. Expression level of each gene was represent by log2(FPKM+1) as colour range from low level (0, blue) to high level(15, red).


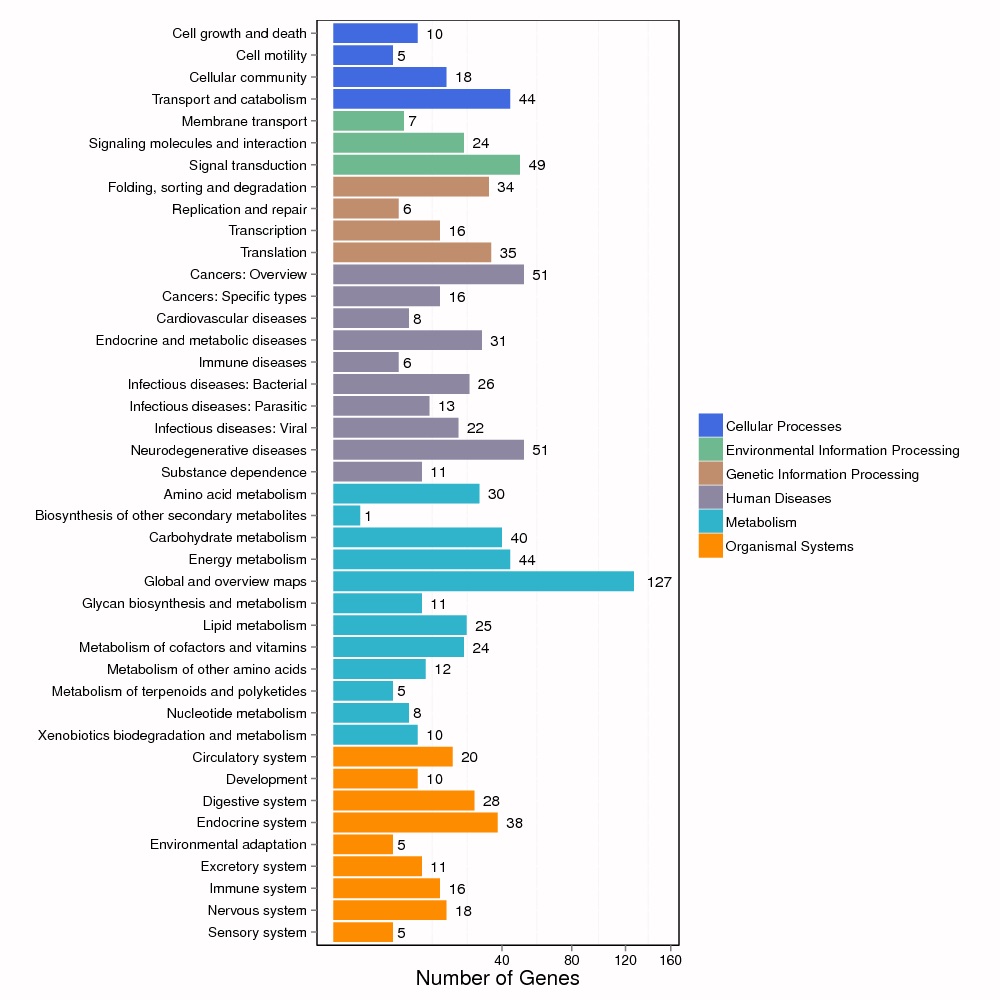


B


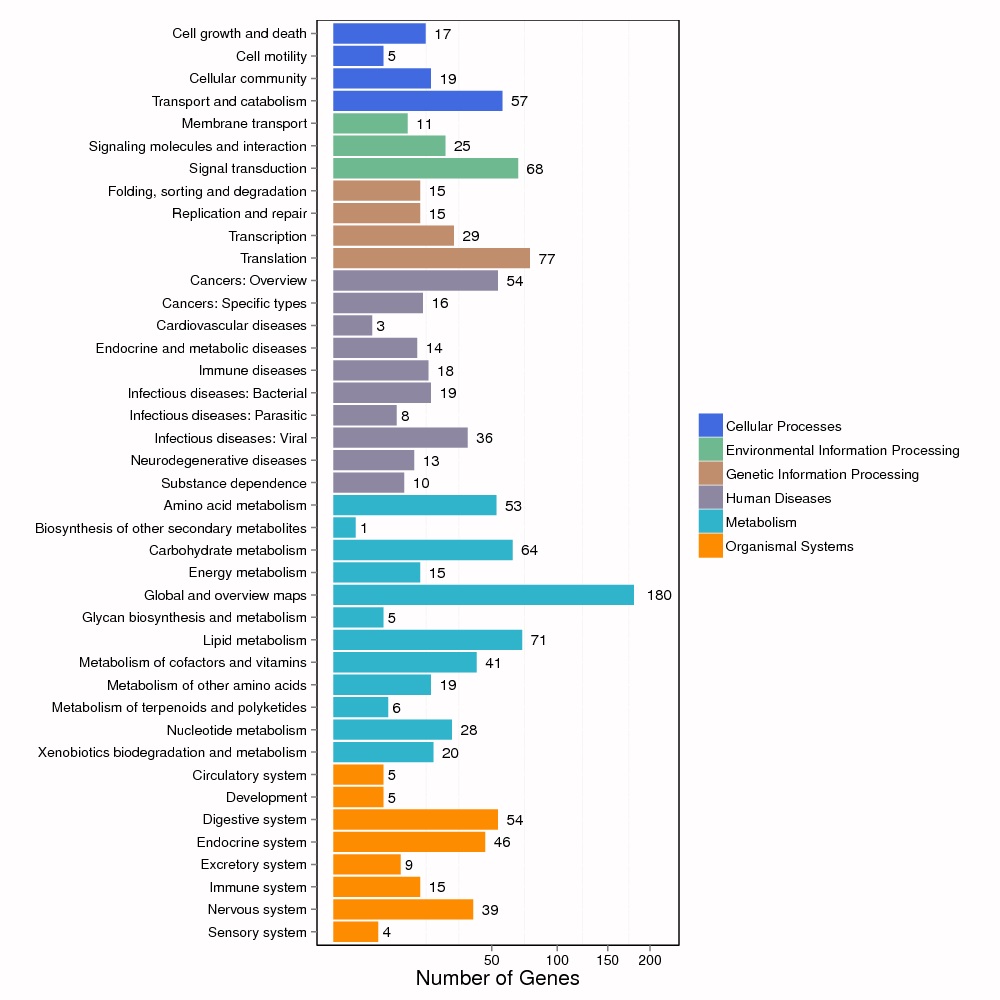


**Fig. S3 KEGG pathway enrichment of differentially expressed genes following with BmCPV infection. A)** differentially expressed genes in fat bodies of infected larvae. **B)** differentially expressed genes in infected midguts.


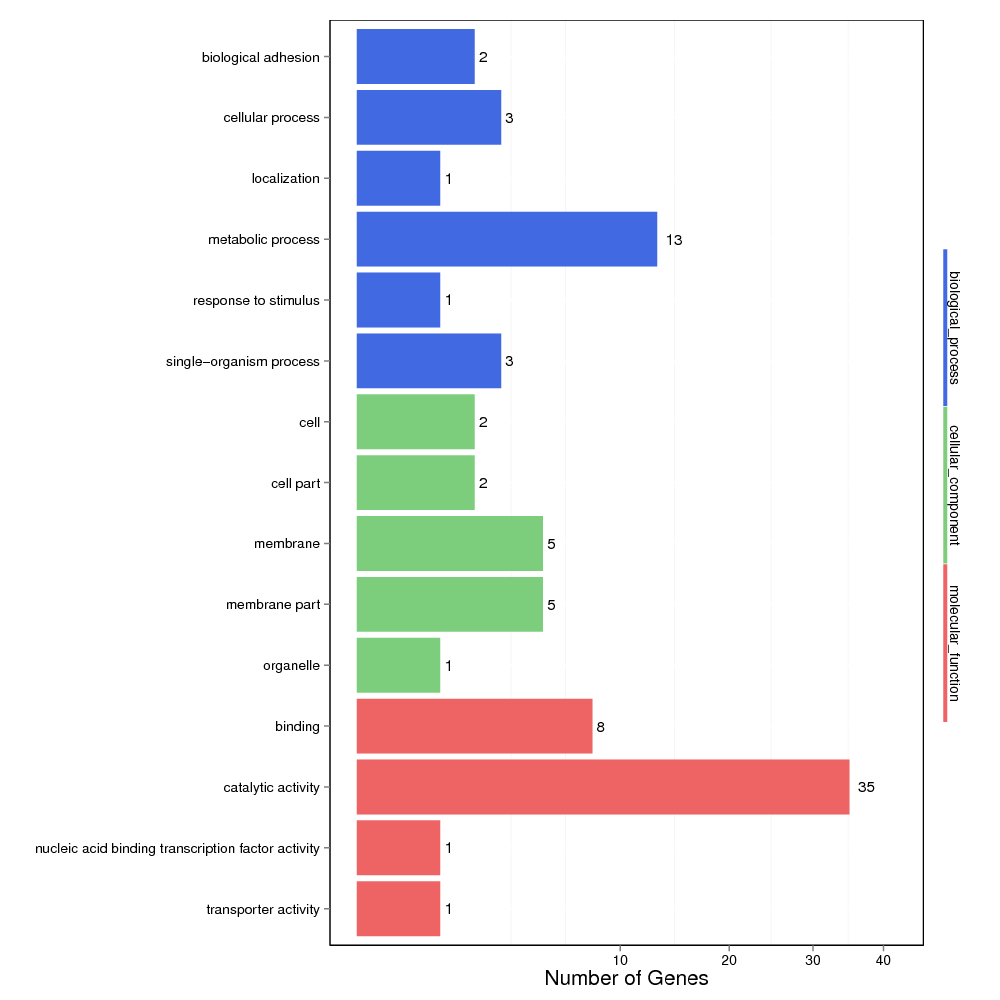


B


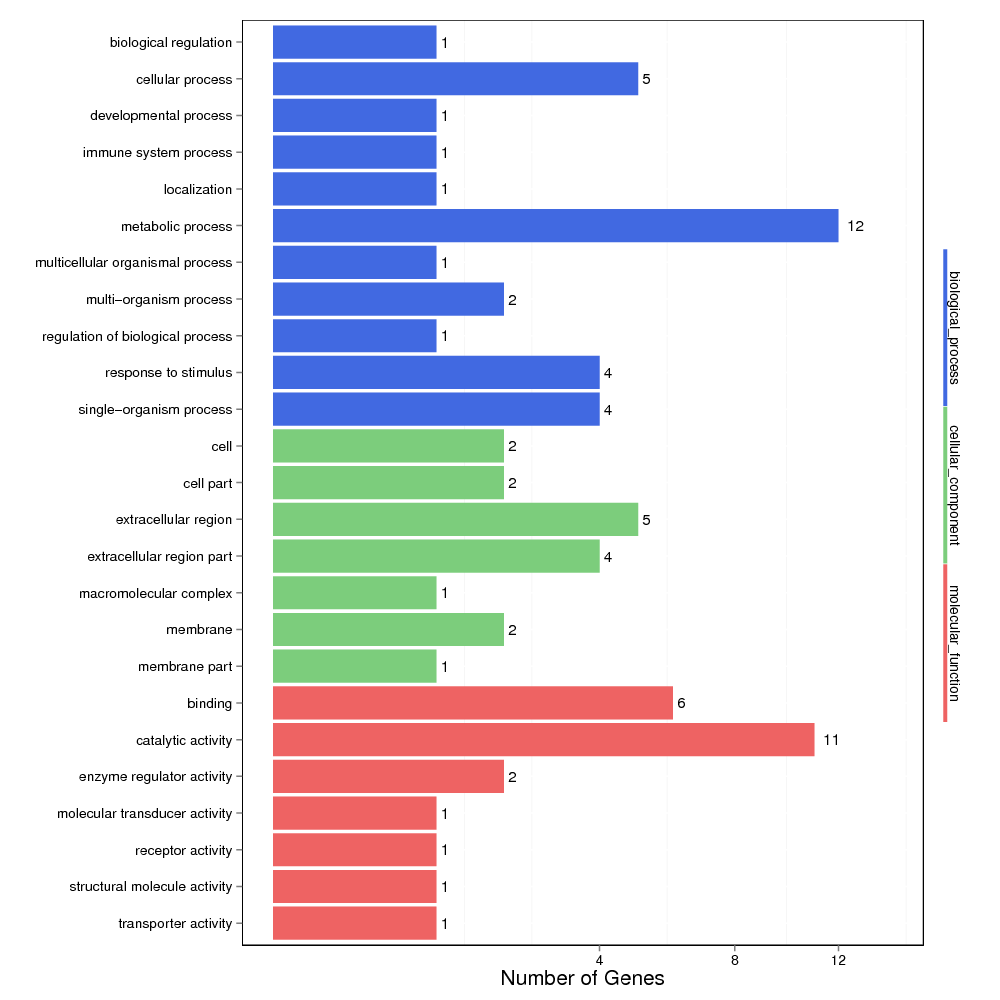


**Fig. S4 Go analysis of both down-regulated and highly or specially expressed genes upon BmCPV expression in A)** midgut or **B)** fat body


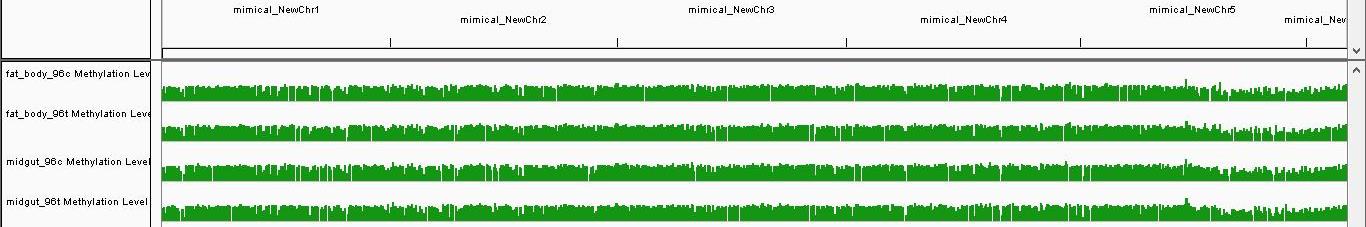


**Fig. S5 The landscape of genomic DNA methylation level of four samples.** Extract the data of all CG site that C methylation level > 0.05 and convert to wig format by perl script for visualization on IGV (Integrative Genomics Viewer). Green vertical line represent the location of each methylated CG site on the genome.


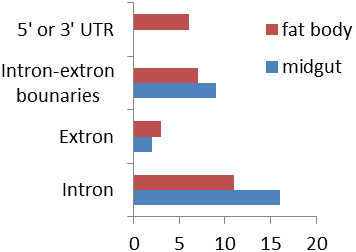


**Fig. S6 DNA methylation distribution of genes that are both differentially expressed and differentially methylated following BmCPV infection.** X-axis represents the number of genes.
